# Elastoïdose à kystes et comédons de Favre et Racouchot

**DOI:** 10.11604/pamj.2013.15.8.2781

**Published:** 2013-05-06

**Authors:** Hayat Bourra, Badreddine Hassam

**Affiliations:** 1 Service de Dermatologie, CHU Ibn Sina, Université Med V, Souissi, Rabat, Maroc

**Keywords:** Maladie de Favre et Racouchot, comédons, Elastoïdose, Favre-Racouchot disease, comedones, elastoidosis

## Image en médicine

La maladie de Favre et Racouchot a été initiallement décrite par Favre en 1932 sous le terme d'élastoïdose nodulaire à kystes et comédons. Elle fait partie des signes de l'héliodermie du visage et du cou, plus fréquente chez l'homme âgé. Caractérisée au début par un épaississement en plaques jaunâtres de la peau, avec élargissement des orifices pilo-sébacés et apparition de comédons, donnant un faux aspect d'acné. Ces lésions siègent de façon symétrique au niveau de la région périorbitaire et temporo malaire, elles peuvent se voir aussi au niveau du nez, du cou et des zones rétroauriculaires, plus rarement au niveau des zones longtemps photoexposées. Au cours de l'évolution, les plaques d'élastose deviennent nodulaires et truffés de comédons regroupés, associés à quelques kystes remplis de kératine. Sur le plan histologique, on retrouve une dégénérescence basophile du collagène, avec accumulation d'un matériel anormal amorphe contenant de l'élastine et présence d'un infiltrat inflammatoire mixte. Le volume des acroinfundibulum et de certains lobules sébacés tend à augmenter constituant les lésions microkystiques et comédoniennes de Favre et Racouchot. Divers traitements peuvent être proposés et combinés: rétinoïdes topiques, isotrétinoïne, curettage, extraction des comédons, dermabrasion, excision chirurgicale, voire Laser CO2. Nous rapportons le cas d'une patiente de 55 ans, suivie pour une néphropathie lupique depuis 10 ans, actuellement sous corticothérapie 20mg/j au long cours, et qui présentait depuis 2ans des lésions kystiques et comédoniennes de la face, peu prurigineuses, évoquant le diagnostic entre autre de lupus ou de Mycosis Fongoïde comédonien, mais dont la biopsie cutanée était en faveur d'une élastoïdose de Favre et Racouchot. L'évolution était favorable sous rétinoïdes topiques mais récidive après 9 mois.

**Figure 1:**
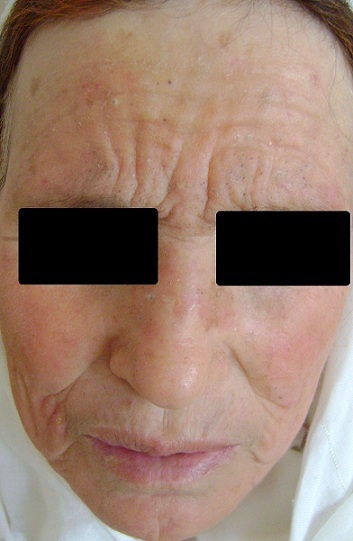
Lésions comédoniennes et kystiques du front, surtout périorbitaires, quelques comédons du nez

